# Exposure to secondhand smoke is associated with poor sleep quality among non-smoking university students in Bangladesh: a cross-sectional survey

**DOI:** 10.1038/s41598-023-43970-6

**Published:** 2023-10-04

**Authors:** Md. Hasan Al Banna, Keith Brazendale, Mohammad Hamiduzzaman, Bright Opoku Ahinkorah, Mohammad Tazrian Abid, M. A. Rifat, Mst. Sadia Sultana, Justice Kanor Tetteh, Satyajit Kundu, Md Shaheenur Rahman Shekhar, Md Khaleduzzaman, Md. Nazmul Hassan

**Affiliations:** 1 Department of Food Microbiology, Faculty of Nutrition and Food Science, Patuakhali Science and Technology University, Patuakhali, 8602 Bangladesh; 2 Nutrition Initiative, Kushtia, Bangladesh; 3 Department of Health Sciences, University of Central Florida, Orlando, USA; 4 School of Health Sciences, The University of Sydney, Sydney, Australia; 5 School of Public Health, University of Technology Sydney, Sydney, NSW Australia; 6 Department of Global Public Health, Karolinska Institutet, 17176 Stockholm, Sweden; 7 Department of Public Health and Informatics, Jahangirnagar University, Savar, Dhaka, Bangladesh; 8 Department of Population and Health, University of Cape Coast, University Post Office, Cape Coast, Ghana; 9 Global Health Institute, North South University, Dhaka, 1229 Bangladesh; 10 Department of English, University of Asia Pacific, Dhaka, 1205 Bangladesh; 11 Department of Environmental Sanitation, Faculty of Nutrition and Food Science, Patuakhali Science and Technology University, Patuakhali, 8602 Bangladesh

**Keywords:** Medical research, Risk factors

## Abstract

Although secondhand smoke (SHS) exposure is predominant in Bangladesh, the adverse effect of SHS exposure on health-related behaviors, such as sleep quality, have remained an under-investigated area of the country’s public health landscape. Therefore, the purpose of this study was to examine the association between SHS exposure and poor sleep quality among non-smoking university students in Bangladesh. A cross-sectional survey was carried out between May and September 2022. SHS exposure (main predictor variable) and other covariates (e.g., age, sex, etc.) were measured using a self-reported questionnaire and sleep quality (outcome variable) was measured via the Pittsburgh Sleep Quality Index. Multiple logistic regression models investigated the association between SHS exposure and poor sleep quality. The study included 390 students (mean age: 22 years, 53.8% male). Approximately 41.8% of the participants reported SHS exposure, and 50.5% had poor sleep quality. Students exposed to SHS were more likely to have poor sleep quality compared to their counterparts (AOR = 1.61; 95% CI 1.01, 2.58). Subgroup analysis revealed poor sleep quality was 2-times higher among male students exposed to SHS than those male students without SHS exposure (AOR = 2.03; 95% CI 1.05, 3.93). No association was found in female students. Findings from this study warrant increased awareness and public health initiatives on the implications of SHS on health behaviors, such as sleep quality, in non-smoking Bangladeshi university students.

## Introduction

Studies have shown sleep quality is a predictor of mental health and wellbeing, and has been linked with an individual’s learning and memory, emotional regulation, and development of brain structures^1–3^. Sleep quality can be determined by assessing several sleep-related outcomes such as sleep latency, sleep duration, sleep efficiency, sleep disturbances, the use of sleeping medication, and daytime dysfunction^4^. Individuals who exhibit poor sleep quality have been linked with adverse health outcomes such as depression, poor physical health, cognitive impairment, depressive symptoms, anxiety, coronary heart disease, hypertension, and a poor quality of life^5–8^. Global health organizations have been concerned with the adverse health effects of insufficient sleep or poor sleep quality in their populations^9^. In 2004, the World Health Organization (WHO) reviewed the evidence on the effects of disturbed sleep on population health. Thus, the causes of poor sleep quality are a priority research area worldwide^10^. Poor sleep quality is becoming more prevalent, with studies reporting up to 60% of the US young adults suffer from poor sleep quality^11,12^, and the prevalence of poor sleep quality increases with age^13^.

Poor quality of sleep has been reported in previous Asian studies^14,15^, including in Bangladesh^16–18^. Studies report that university students have more sleep disturbances and poor sleep quality compared to other demographic groups^19,20^. A variety of factors have been explored as potential predictors of poor sleep quality in university students such as extensive academic obligations, lifestyle choices (more flexibility and freedom), and behavioral factors (such as smartphone/internet addiction). However, studies examining other notable factors—such as secondhand smoking, stress, anxiety, chronic health conditions, bed-time social media use, poor sleep habits, noise, and Apnea^6,21,22^—are limited.

Bangladesh is reported to have a high prevalence of exposure to secondhand smoke (SHS) due to the cheap cost and accessibility of cigarettes^23,24^. In Bangladesh, the prevalence of secondhand smoking was found to be 39.1% for adults and 31.1% for children aged 13 to 15 in earlier studies conducted in 2013 and 2017^23,25,26^. In addition, the prevalence of smoking among university students in Bangladesh is 60.2%, thus, increasing the likelihood of exposure to SHS^27^. Currently, there is a lack of understanding and awareness on the adverse effect of SHS^28^. Although SHS exposure in Bangladesh has been strongly linked with gender, age, literacy, household incomes, and number of smokers in the family^29^, the adverse consequences of secondhand smoking on university students’ sleep quality have remained an under-investigated area in the country’s public health landscape. International studies exploring this relationship do exist. In China, for example, studies have reported a significant relationship between SHS exposure and sleep quality among young adults and children^2,30^. In Turkey, approximately two-thirds of adult students sampled from a university reported SHS exposure as one of the key contributors of poor sleep quality^22^. Similarly, in Japan, a study reported that young adult females who had been exposed to SHS had worse sleep quality than those who did not^31^.

There is a dearth of information available to guide public health policy and decision making in Bangladesh. While the prevalence of smoking and exposure to SHS is high in Bangladesh^25–27^, there is, however, limited research on how this may impact health-related behaviors, such as sleep quality. Therefore, the purpose of this study is to examine the association between SHS exposure and sleep quality in a large sample of non-smoking Bangladeshi university students. We hypothesize that SHS exposure is associated with poor sleeping quality among non-smoking university students. Findings from this study have the potential to inform future interventions to improve health outcomes in Bangladeshi university students.

## Materials and methods

### Study design and ethics

A cross-sectional survey was carried out between May and September 2022. This survey was conducted in accordance with the Declaration of Helsinki, and the study design was reviewed and approved by the Research Ethical Committee (REC) of the Department of Environmental Sanitation, Patuakhali Science and Technology University (PSTU), Bangladesh (approval number: ENS:09/04/2022:05). Written informed consent was taken from the study participants after assuring the anonymity and confidentiality to their data.

### Study setting

Two public universities in Bangladesh—PSTU and University of Barishal (BU)—were selected as sites for the current investigation. Both universities are situated in the Barishal division, a southern coastal region of Bangladesh. PSTU has eight residence dormitories (“halls”) and eight colleges, with approximately 4000 students. BU has four residence halls and six colleges, with around 5000 students. The PSTU campus is about 20 km north of the city of Patuakhali district, and about 38 km south of Barishal city.

### Study sample

The following criteria had to be met by potential participants to be eligible as participants: (i) 18 years of age or older; (ii) self-reported non-smoking students; and (iii) being a full-time student. Initially, the following screening question was asked: “Have you smoked in the past month?” (Yes vs. No). The students who responded “no” (i.e., non-smokers) were invited to participate in the study. The students who smoke were excluded from the study. Moreover, participants were excluded if they reported medical or mental health conditions.

The sample size was calculated using the single population proportion test by considering the following assumptions: (i) 66.6% prevalence of poor sleep quality among Bangladeshi public university students was used (*p* = 0.67) based on the previous investigation^21^, (ii) 95% confidence (Z = 1.96) and (iii) 5% margin of error (d = 0.05). The calculation computed was as follows: ${\text{Minimum}}\;{\text{sample}}\;{\text{size}},\;n = \frac{{z^{2} \times p \times (1 - p)}}{{d^{2} }} = \frac{{(1.96)^{2} \times 0.67 \times (1 - 0.67)}}{{(0.05)^{2} }} = 339.75 \approx 340$.

An optimal sample of 374 participants was obtained by using a 10% non-response rate to account for the potential compensation of non-responses. Previous Bangladeshi study also accounted 10% non-responses of university students for calculating optimal sample size^32^. Finally, a total of 390 participants were included in the study. The participants were chosen using a cluster sampling technique with two stages. First, 500 rooms were selected conveniently from each block of ten dormitories (e.g., block 1, block 2, and so on). Second, up to two students were drawn at random from each of the selected rooms (approximately 4 students per room).

### Study protocol

In-person interviews were conducted to obtain study data from the respondents. A paper-based questionnaire was used. The data collection team was composed of four interviewers (2 males and 2 females) who were university students. Interviewers were trained by the principal investigator of this study on the measures used, interview techniques, sampling approach, participants’ consent process, and eligibility criteria of the study. As data were gathered from students living in university dormitories, male interviewers visited the male dormitories for data collection, and vice-versa. Following permission from the room members, the interviewers entered the selected rooms and invited them to participate voluntarily after explaining the purposes of the study. Interested students were assessed for study eligibility, and once all criteria had been met, the student participated in the structured interview schedule. Each interview lasted between 15 to 20 min.

### Study measures

Sleep Quality. The sleep quality of students, the outcome variable of this study, was evaluated using the 19-item Pittsburgh Sleep Quality Index (PSQI) questionnaire^33^. Each question and response option was read to the participant and completed by the interviewer. The PSQI is categorized into seven components (i.e., subjective sleep quality, sleep latency, sleep duration, habitual sleep efficiency, use of sleep medication, and daytime dysfunction), which assess sleep quality and disturbances over the last month. The score of each component ranges from 0 to 3. The overall PSQI score of sleep quality is created by summing the seven component scores, which range from 0 to 21. A PSQI score greater than 5 was the threshold for poor sleep quality. The PSQI scale is widely used among different sub-populations^34^, including Bangladeshi students^16,21,35^. In this study, Cronbach's alpha of the scale is 0.736, which indicates an acceptable level of internal consistency^36^.

Secondhand Smoke Exposure. In accordance with other studies exploring exposure to SHS^8^, the SHS exposure of self-reported non-smoking individuals was assessed by asking the following single question (Yes vs. No): “Are you exposed to secondhand smoke in a confined environment (such as at a room, work, or hangout place like a tea stall) for more than 15 min at a time within a week?” Secondhand smoking is referred to as a passive inhalation of “a tobacco side-stream smoke” throughout this study.

Covariates. Participants’ sociodemographic and behavioral characteristics were captured. Participants' age and family income were obtained as a continuous measure which were categorized into different groups for data analysis. Participants' gender, academic background, religion, and marital status were also included. Moreover, this study included participants' behavioral information such as self-rated body mass index status (underweight, normal weight, or overweight/obese), bed-time social media use (no, < 1 h and ≥ 1 h), laptop ownership (yes and no), and physical activity level (inactive, moderate, and regular).

### Statistical analyses

Both analytical and enumerative statistics were calculated to analyze the data. Categorical variables were summarized as responses, percentages, and bar diagrams. Means and standard deviations were computed for continuous variables. A chi-square test was performed to show the distribution of sleep quality status across the independent variables.

A multi-variable logistic regression analysis was conducted to identify the association between SHS exposure and poor sleep quality. In addition, separate multi-variable logistic regression models were computed to explore this relationship by male versus female. All covariates were incorporated in the adjusted model to assess the estimated adjusted impacts of SHS exposure on poor sleep quality (see Table 1). The variance inflation factor (VIF) was used to test for multicollinearity among the independent variables before running the regression analyses (see Table 1). All the adjusted regression models were selected based on the Hosmer and Lemeshow goodness of fit test (see Table 1). The strength of the association between SHS exposure and poor sleep quality was presented as odds ratios with 95% confidence intervals and standard errors for both the adjusted and unadjusted regression models. The cut-off value for statistical significance was set at ≤ 0.05. All analyses were computed using the Statistical Package for the Social Sciences (SPSS, IBM version 23.0, Armonk, NY, USA).

**Table 1:** The output of multicollinearity test and adjusted regression model fitness test, and summary of the variables adjusted for assessing the estimated adjusted effects of SHS exposure on poor sleep quality.

Features	Model 1: (all participants)	Model 2: (only males)	Model 3: (only females)
Multicollinearity test	Mean VIF = 1.141 (< 10)	Mean VIF = 1.121 (< 10)	Mean VIF = 1.099 (< 10)
	Minimum = 1.062	Minimum = 1.024	Minimum = 1.035
	Maximum = 1.334	Maximum = 1.256	Maximum = 1.158
Hosmer and Lemeshow test	Chi-square (df) = 11.451(8)	Chi-square (df) = 11.936 (8)	Chi-square (df) = 8.064 (8)
	*P* value = 0.177	*P* value = 0.154	*P* value = 0.427
Adjusted variables with SHS exposure	Gender, age, monthly family income, religion, educational background, marital status, self-perceived BMI status, bed-time social media use, own a laptop and self-reported physical activity level	Age, monthly family income, religion, educational background, marital status, self-perceived BMI status, bed-time social media use, own a laptop and self-reported physical activity level	Age, monthly family income, religion, educational background, marital status, self-perceived BMI status, bed-time social media use, own a laptop and self-reported physical activity level

## Results

The sample consisted of 390 non-smoking students between the ages of 18 and 27 years (mean age: 22.43 years, SD: 1.54), with males making up 53.8% (n = 210) of the sample. More than one-third of the students (36.4%) were studying biological sciences (see Table 2).

**Table 2:** Bivariate analysis shows the distribution of sleep quality based on participants’ characteristics (N = 390).

Variables	Total, n (%)	Sleep quality, n (%)		Chi-square (*χ*^2^*)*statistic	
		Good	Poor	*χ2* value (df)	*P* value
Sleep quality		193 (49.5)	197 (50.5)		
Gender				0.353 (1)	0.553
Male	210 (53.8)	101 (48.1)	109 (51.9)		
Female	180 (46.2)	92 (51.1)	88 (48.9)		
Age (years)				1.353 (2)	0.508
18–21	105 (26.9)	56 (53.3)	49 (46.7)		
22–25	272 (69.7)	132 (48.5)	140 (51.5)		
> 25	13 (3.4)	5 (38.5)	8 (61.5)		
Monthly family income (BDT)				2.209 (2)	0.331
≤ 20,000	145 (37.2)	78 (53.8)	67 (46.2)		
20,001–40,000	159 (40.8)	72 (45.3)	87 (54.7)		
> 40,000	86 (22.1)	43 (50.0)	43 (50.0)		
Religion				0.136 (1)	0.712
Muslim	332 (85.1)	163 (49.1)	169 (50.9)		
Hindu	58 (14.9)	30 (51.7)	28 (48.3)		
Educational background				2.024 (4)	0.731
Engineering	25 (6.4)	14 (56.0)	11 (44.0)		
Health science	77 (19.7)	35 (45.5)	42 (54.5)		
Biological science	142 (36.4)	74 (52.1)	68 (47.9)		
Business studies	31 (7.9)	13 (41.9)	18 (58.1)		
Others	115 (29.5)	57 (49.6)	58 (50.4)		
Marital status				0.219 (1)	0.640
Single	355 (91.0)	177 (49.9)	178 (50.1)		
Married	35 (9.0)	16 (45.7)	19 (54.3)		
Self-perceived BMI status				3.848 (2)	0.146
Underweight	61 (15.6)	36 (59.0)	25 (41.0)		
Normal weight	256 (65.6)	118 (46.1)	138 53.9)		
Overweight	73 (18.8)	39 (53.4)	34 (46.6)		
Bed-time social media use				1.254 (2)	0.534
No	43 (11.0)	22 (51.2)	21 (48.8)		
< 1 h	136 (34.9)	72 (52.9)	64 (47.1)		
≥ 1 h	211 (54.1)	99 ((46.9)	112 (53.1)		
Own a laptop				0.031(1)	0.860
Yes	222 (56.9)	109 (49.1)	113 (50.9)		
No	168 (43.1)	84 (50.0)	76 (50.0)		
Self-reported physical activity level			1.253 (2)	0.534	
Physically inactive	67 (17.2)	37 (55.2)	30 (47.2)		
Moderate activity	180 (46.2)	85 (44.8)	95 (52.8)		
Regular activity	143 (36.6)	71 (49.7)	72 (50.3)		
Exposed to secondhand smoke				3.938 (1)	**0.047**
Yes	163 (41.8)	71 (43.6)	92 (56.4)		
No	227 (58.2)	122 (53.7)	105 (46.3)		

BDT = Bangladeshi taka (currency), BMI = body mass index, Bolded value indicates statistical significance (*p* < 0.05).

Of the total participants, 41.8% of them reported being exposed to SHS, and half of the students (50.5%) reported poor sleep quality according to the PSQI. Results from the chi-square test showed that SHS exposure was significantly associated with sleep quality (chi-square, *χ2* = 3.938, *p* = 0.047) (see Table 2).

**Figure 1:**
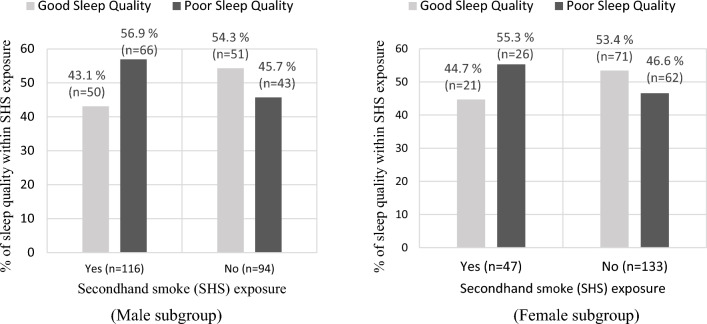
Descriptive presentation of male and female students’ status of sleep quality (good vs. poor) within the category of secondhand smoke exposure (yes vs. no).

Figure 1 depicts that the prevalence of poor sleep quality was higher among male (56.9% vs. 45.7%) and female (55.3% vs. 46.6%) participants with SHS exposure than their counterparts. The chi-square analysis found no significant association between SHS exposure and sleep quality in the male (*χ*^2^ = 2.587, *p* = 0.108) and female subgroup (*χ*^2^ = 1.053, *p* = 0.305).

Overall, the adjusted multiple logistic regression models revealed that students who were exposed to SHS were more likely to have poor sleep quality compared to their counterparts (adjusted odds ratio, AOR = 1.61; 95% CI: 1.01, 2.58). For the male subgroup, poor sleep quality was 2 times higher among students with SHS exposure than those without SHS exposure (AOR = 2.03; 95% CI: 1.05, 3.93). The association between SHS exposure and poor sleep quality was insignificant in the female subgroup (see Table 3).

**Table 3:** Multiple logistic regression models demonstrating the link between secondhand smoke exposure and poor sleep quality among non-smoking university students in Bangladesh.

Subject	Variable(s)	Unadjusted model					Adjusted model †				
		COR	SE	Wald	95% CI	*P*	AOR	SE	Wald	95% CI	*P*
Model 1: All (n = 390)	SHS exposure										
	Yes	1.51	0.21	3.92	1.00–2.26	**0.048**	1.61	0.24	3.96	1.01–2.58	**0.047**
	No	Ref					Ref				
Model 2: Males (n = 210)	SHS exposure										
	Yes	1.57	0.28	2.58	0.91–2.71	0.109	2.03	0.34	4.45	1.05–3.93	**0.035**
	No	Ref					Ref				
Model 3: Females (n = 180)	SHS exposure										
	Yes	1.42	0.35	1.05	0.73–2.77	0.306	1.44	0.39	0.88	0.67–3.09	0.349
	No	Ref					Ref				

SHS = secondhand smoke, COR = crude odds ratio, SE = standard error, CI = confidence interval, *p* = probability value, AOR = adjusted odds ratio, Ref = reference group. Bolded values indicate statistical significance (*p* < 0.05).

^†^The summary of adjusted model 1, 2 and 3 is presented in Table 1.

## Discussion

This cross-sectional study investigated the association between SHS and poor sleep quality among non-smoking university students in Bangladesh. Findings from this study reveal that SHS exposure was significantly associated with poor sleep quality in Bangladeshi non-smoking university students. A subgroup analysis revealed that this association was significant only for male, and not female, student study participants. These findings suggest that university authorities should design and implement public health interventions including tobacco control policies, behavioral change models that incorporate sleep hygiene to reduce smoking and improve the students’ sleep quality, especially for male students.

The major finding that SHS exposure is significantly associated with poor sleep quality is consistent with findings from some previous studies in different population groups, including children^2,37^, adolescents^2,38^, university students^22^, and adults^6,31^. Although similar findings are widely reported in literature, a study in USA found no statistically significant relationship between poor sleep quality and SHS among the general population^39^. A mixed result was observed by a recent meta-analysis of observational study, showing a statistically significant association between SHS exposure and poor sleep quality among adolescents but not in adult subgroups^34^. A plausible explanation of this relationship could be adolescents are more likely than adults to have a higher stress response^40^, which might work in conjunction with nicotine exposure to cause more sleep interruptions. Additionally, variations in results may result from sample size, analytical techniques, geographical and cultural variety, and demographic differences. Our finding suggests the need for university campuses in Bangladesh to consider implementing protective practices for non-smoking students (e.g., separate smoking areas, air purifiers in common spaces occupied by students, or dormitory rooms), and to offer assistance to students who smoke, such as programs to help/encourage smoking cessation.

Male study participants reporting SHS exposure were twice as likely to report poor sleep quality compared to males without SHS exposure. This finding could be explained by the high smoking frequency of males compared to females in Bangladesh^41^. Previous studies have reported males were approximately 13 times more likely to smoke than females in Bangladesh^41^. This pattern has also been observed among Bangladeshi university students^27,42^, for instance, with 49.1% of male students identifying as smokers compared to 2.4% of female students in PSTU (Patuakhali, Bangladesh)^42^. Other underlying factors could also contribute to the significant association between poor sleep quality among male non-smoking students and their SHS exposure. For example, the likelihood of having a friend who smokes is higher for male students compared to their female counterparts. Thus, peer influence may moderate this relationship through other mechanisms such as regular sleep disturbances. Non-smoker students may modify sleep habits to stay awake with their friends who smoke, who already have sleep disturbances due to their active smoking behavior^43^. Although no significant association between SHS and poor sleep quality among females was found, a high proportion (55.3%) of females who were exposed to SHS reported poor sleep quality. This re-emphasizes the need for a closer look at the problem of SHS in educational institutions in Bangladesh and a concerted effort by various stakeholders to curb the problem.

In the adjusted model, no other significant associations were found between covariates and sleep quality. One possible reason could be the close to equal distribution of the frequency of the covariates such as gender, age, monthly family income, marital status, self-reported BMI status, and self-reported physical activity level of the participants. This implies that poor sleep quality is an issue among half of the university students in Bangladesh regardless of how they are categorized. Findings from previous studies also support this statement. For example, several studies have reported the prevalence of poor sleep quality among Bangladeshi university students ranges from 48.5% to 66.6%^16,17,21^. The evidence pertaining to gender differences in sleep quality among students is contradictory. Ahmed et al.^21^ found that female university students were 4.1 times more likely to report having poor sleep quality than male university students, whereas a study by Mamun et al.^16^ found poor sleep quality was significantly associated with being male in a sample of 450 students from Bangladesh. In contrast to this study, factors associated with poor sleep quality have mainly focused on other environmental and behavioral predictors such as internet use, social media use, and direct smoking behavior. In the present study, it is plausible that other environmental or behavioral factors could be contributing to poor sleep quality in students, outside of SHS exposure. Poor dietary habits, noisy sleep environments on university premises, poor sleep hygiene practices (e.g., long periods of exposure to screen time before bed), and an increase in social activity as students experience a major life transition when they leave the family homes to begin studying at the university level. There is a paucity of evidence that has explored these additional factors in relation to sleep quality in university students, thus, future studies are needed to better understand the key determinants of poor sleep quality in students from both a quantitative and qualitative standpoint.

The present study excluded smoker participants. However, it must be noted smokers are more vulnerable to adverse sleep health compared to non-smokers. A study conducted in China among the general population (n = 26,282; participants ≥ 12 years old) showed that sleep disturbances and poor sleep quality were more prevalent among cigarette smokers than nonsmokers^44^. A recent systematic review and meta-analysis of prospective studies (age ≥ 12 years) reported smoking is related to a higher risk of developing sleep-related issues (such as insomnia, sleep disturbances, sleep complaints, sleep difficulty, etc.)^45^. The meta-analysis demonstrated that smokers were 1.47 times more likely to experience sleep-related problems than non-smokers. Based on the literature and the present study’s findings, both active and passive smoking have a deleterious effect on sleep and sleep-related disorders. Further follow-up studies, which include both smokers and non-smokers, and mental health conditions like depression, anxiety and stress, are recommended to identify the causal factors of poor sleep quality among students.

### Strength and limitations

Strengths of this study include the use of a validated measure of sleep quality and the large sample size. In addition, this study is one of the first to assess the association between exposure to SHS and poor sleep quality in Bangladeshi non-smoking students. This is significant as Bangladesh has a high prevalence of smoking and SHS exposure among students, and, thus, the health behavior implications of this must be understood. This study has limitations that must be acknowledged. The cross-sectional nature of the study design does not allow causality to be established between SHS exposure and sleep quality. The majority of students in our sample were studying biological sciences, therefore, generalizability of findings may be limited. Since this study covered only two public universities from one division of Bangladesh (Barisal division), our findings do not generalize to other contexts and populations such as private universities or other demographic groups. There is always the possibility of social desirability biases when using self-report measures, thus findings must be interpreted with caution. The screening of SHS exposure might be overestimated or underestimated due to the self-reported nature of the SHS measure.

## Conclusion

In summary, results from this cross-sectional study revealed an association between SHS exposure and poor sleep quality among non-smoking students sampled from two public universities in Bangladesh. Furthermore, a significant association was found for male, but not female, students. These findings suggest that SHS is a potential contributor of poor sleep quality among non-smoking university students in Bangladesh. Future longitudinal research is needed to determine causality, however, there are several tangible steps that university campuses in Bangladesh can take to promote a healthier campus environment and protect their student body. For example, implementing protective practices for non-smoking students (e.g., separate smoking areas) and offering assistance to students who smoke, such as programs to help/encourage smoking cessation, could be a positive step forward. Further, universities could take advantage of the portion of the Smoking and Tobacco Products Usage (Control) (Amendment) Act, 2013 that allows sub-national regulations, that are stricter than national law, to enact policies and bi-laws on university campuses to reduce the risk of SHS exposure especially for non-smoking students in Bangladesh. Collectively, these practices may reduce the health and disease burden related to smoking and SHS exposure in the Bangladeshi student population.

## Data Availability

The datasets used and/or analyzed during the current study are available from the corresponding author on reasonable request.
